# What quantifies good primary care in the United States? A review of algorithms and metrics using real-world data

**DOI:** 10.1186/s12875-023-02080-y

**Published:** 2023-06-24

**Authors:** Yun Wang, Jianwei Zheng, Todd Schneberk, Yu Ke, Alexandre Chan, Tao Hu, Jerika Lam, Mary Gutierrez, Ivan Portillo, Dan Wu, Chih-Hung Chang, Yang Qu, Lawrence Brown, Michael B. Nichol

**Affiliations:** 1 School of Pharmacy, Chapman University, Irvine, US; 2 Gehr Center for Health Systems Science and Innovation, Keck School of Medicine, University of Southern California, Los Angeles, US; 3 Department of Clinical Pharmacy Practice, School of Pharmacy & Pharmaceutical Sciences, University of California, Irvine, US; 4 Department of Geography, Oklahoma State University, Stillwater, US; 5 Department of Clinical Research, Faculty of Infectious and Tropical Diseases, London, School of Hygiene and Tropical Medicine, London, UK; 6 Program in Occupational Therapy, Department of Medicine, and Department of Orthopaedic Surgery, Washington University School of Medicine, St. Louis, MO US; 7 Sol Price School of Public Policy, University of Southern California, Los Angeles, US

**Keywords:** Primary care, Quality, Electronic health records, Claims data, Metrics, Algorithms

## Abstract

Primary care physicians (PCPs) play an indispensable role in providing comprehensive care and referring patients for specialty care and other medical services. As the COVID-19 outbreak disrupts patient access to care, understanding the quality of primary care is critical at this unprecedented moment to support patients with complex medical needs in the primary care setting and inform policymakers to redesign our primary care system. The traditional way of collecting information from patient surveys is time-consuming and costly, and novel data collection and analysis methods are needed. In this review paper, we describe the existing algorithms and metrics that use the real-world data to qualify and quantify primary care, including the identification of an individual’s likely PCP (identification of plurality provider and major provider), assessment of process quality (for example, appropriate-care-model composite measures), and continuity and regularity of care index (including the interval index, variance index and relative variance index), and highlight the strength and limitation of real world data from electronic health records (EHRs) and claims data in determining the quality of PCP care. The EHR audits facilitate assessing the quality of the workflow process and clinical appropriateness of primary care practices. With extensive and diverse records, administrative claims data can provide reliable information as it assesses primary care quality through coded information from different providers or networks. The use of EHRs and administrative claims data may be a cost-effective analytic strategy for evaluating the quality of primary care.

## Background

In the United States, primary care physicians (PCPs) have the essential responsibilities of referring their patients to appropriate specialists, ensuring the coordination of clinical care and other medical resources, and helping to detect, treat and prevent illness. Given the critical role of PCPs in healthcare, efficient quality assessment of primary care remains indispensable. The U.S. Agency for Healthcare Research and Quality (AHRQ) develops patient experience and satisfaction surveys to determine the quality of primary care [1]. However, the significant problems with these subjective, self-reported measurements are that they are time- and cost-consuming [2]. It is arduous to produce a representative yet standardized survey across the country to measure patients’ perspectives regarding primary care quality and allow for meaningful comparisons among peers. In contrast, big data in healthcare is an easier and faster data organization that could alleviate time and money consumption. Big data in healthcare includes, but is not limited to, electronic health records (EHRs), claims data collected by payor records, medical imaging, genomic sequencing, and eHealth data [3]. Objective metrics based on existing EHRs and administrative claims data demonstrate the tremendous possibility of examining primary care effectively.

Measuring the performance of PCP care continues to be an important yet challenging part of clinical research and quality improvement due to the lack of the “common vocabulary [4]”. In the early period of primary care establishment, Barbara Starfield emphasized the four different responsibilities of high-quality primary care services, which include “first-contact accessibility, continuity, comprehensiveness, and coordination [5].”

“First contact accessibility” describes the capacity of a primary care system to serve as the first contact person, gatekeeper to the health system, and coordinator of care. To promote first-contact accessibility and person-centeredness, it is important to focus efforts on where people currently seek primary care services and who is the most likely responsible PCP. “Continuity of Care,” as a key pillar of an effective healthcare system, is defined as seeing the same PCP over time [6]. “Comprehensiveness” overlaps the scope of practice, sites of care depth, and breadth of conditions managed by the PCP [7], including prevention and wellness and acute, chronic, and comorbid condition management. “Coordination” refers to the holistic organization of patient-physician interaction among multiple healthcare providers [8], for example, the specialists and PCPs. No consensus definition has fully evolved for the definition of “comprehensiveness” and “coordination” [9]; however, they are both based mainly on the “process” measures of primary care.

## Type of data resources, and strength and weakness of these data sources

EHRs and administrative claims data represent two different sets of real-world healthcare information resources. The EHRs reflect the practice pattern by recording and managing patient care, while claims data track the patient’s utilization of services based on the billing and payment of health services. The use of EHRs and claims data to analyze patients’ PCP encounters requires comprehensive computerization of primary care practices. Along with national level efforts following the Health Information Technology for Economic and Clinical Health Act 2009 [52], EHR audits may have an immense potential for a large number of digital medical records to describe the workflow of PCP encounters quantitatively. Administrative claims data, based on large and diverse records from the integrated cloud and on-premises data warehouses, could include billing codes for patients' primary care services from different providers or networks and reduce the selection bias from a single provider or network. The use of administrative claims data can also provide a comprehensive view of a patient’s encounters with providers (e.g., place and date of service, diagnosis history and codes, treatment, and PCP types), beneficiary benefit coverage, pharmacy records, procedures, and performance of laboratory tests.

## Measurements of PCP performance

A wide range of quality indicators and algorithms have been proposed to evaluate these aspects of primary care (Table 1): 1) identifying an individual’s likely responsible PCP, 2) process measures, and 3) the continuity and regularity of care. In this review paper, we examined and compared the existing algorithms and metrics and discussed the strengths and limitations of EHR and claims data, as well as specific case examples. The structural knowledge of the measurements can help researchers identify and make the most appropriate choices among different conceptions and methodological strategies.

**Table 1:** Algorithms and metrics as quality indicators of primary care

Measurements	Algorithms
Identifying an individual’s likely PCP [12]: To find out the designated primary care physician	1) Plurality provider: Provider who billed the greatest number of Evaluation & Management (E&M) visits in a year for the beneficiary (includes specialists) 2) Majority provider: Provider who billed for the plurality of E&M visits (must be > 50% of all visits)
Process quality [15]: Quality measurement and reporting the comprehensiveness of care [7] (both the scope of services offered and the depth and breadth of conditions managed by the primary care team)	1) Appropriate-care-model composite score for preventive care [15–17]: The five measures were the appropriate-care-model composite measures for diabetes, medication management, and depression. (A composite measure combines the individual measures of care needed for a condition. In the case of diabetes, the composite measure includes the receipt of cholesterol screening, eye exams, urine protein screening, and an HbA1c test, which measures blood glucose.) 2) First contact [18, 19]: Time from discharge to the first PCP visit 3) Negligent adverse events [68]: Determinations of negligence were based on peer reviews 4) Documentation of lifestyle counselling from PCP notes in EHR [13]: Settled claims for negligent adverse events are an expression of patients’ experiences of medical errors and provide a useful insight into errors in primary care 5) Medication intensification [13]: Defined as initiation of a new medication or an increase in the dose of an existing medication 6) Duration of consultation time [20, 69] 7) “No-shows” and “same-day” cancellations, “after-hours” care availability [34] 8) Prevention Quality Indicators (PQI) [70]: episodes that may be potentially avoided through the timely receipt of primary or preventive care, including diabetes short-term complications admission rate, perforated appendix admission rate, diabetes long-term complications admission rate, etc 9) Care density [70] measures the extent of ‘patient-sharing’ among an individual’s ambulatory providers. The numerator of care density is the sum of shared patients among each pair of a patient’s outpatient doctors, and the denominator is the total number of pairs of outpatient doctors that a patient sees 10) Geographic accessibility to primary care providers [30, 31]: spatial accessibility to primary care service (i.e., PCPs/10,000 population, availability of PCPs within a global service catchment of 30-min drive time)
Continuity index [41]: Degree of coordination required between different providers during an episode or	1) Continuity of Care (COC) index [71]:$COC-\frac{\sum_{i=1}^Pn_i^2-N}{N\left(N-1\right)}$ , P = total number of providers, P = total number of providers, N = total number of visits, n_i_ = number of visits per provider i., ni = number of visits per provider i. This index weights both the frequency of visits to each caretaker and the dispersion of visits between caretakers. Index values range from 0 (each visit made to a different physician) to 1 (all visits made to a single physician) 2) Number of Providers Seen (NOP) [72]: Number of providers with whom the patient had contact in a defined time interval (e.g., one year) 3) Sequential Continuity Index (SECON) [44, 73]: Measures the number of visits made to the caretaker whom the patient saw in the most recent visit. This index is useful for assessing the need to share information among caretakers. Index values range from 0 (every visit made to a physician other than the physician seen in the previous visit) to 1 (all visits made to a single physician): $SECON=\frac{\varnothing 1+\dots +\varnothing n-1}{N-1}$, where ∅ = 1 if visit i & i + 1 are to the same provider and ∅ = 0 if otherwise, and N = total number of visits 4) Likelihood of Sequential Continuity Index (LISECON) [74]: the likelihood that SECON is greater than would occur if distribution of practitioners across sequential visits were random 5) Likelihood of Continuity Index (LICON) [75]=$1-\sum_{i=1}^{k}{p}_{n}i+\frac{i}{M}{p}_{n-1} (i)$ where ${p}_{n}i=\frac{M-\left[I-1\right]}{M}{p}_{n-1}\left(i-1\right)+\left(\frac{i}{M}\right){p}_{n-1}i;$ N = total number of visits; n_i_ = number of visits to i-th provider and Pn(k) if the probability of seeing k different providers in n visits; M = total number of providers 6) Herfindahl Index (HI) [76]=$\sum_{i=1}^{p}{(\frac{ni}{n})}^{2},$ where p = total number of providers, n = total number of visits during episode ni = number of visits to provider i,cj = indicator of sequential visits to same providers; equal to 1 if visits j and j + 1 are to the same provider, 0 otherwise 7) Modified Continuity Index (MCI) [77] = $\frac{\sum_{i=1}^{k}ni /k}{\sum_{i=1}^{k}pi /k}$, where n_i_ = no of visits and p_i_ = total number of providers seen by patient i in population k during a defined time interval 8) Modified, Modified Continuity Index (MMCI) [78]: $MMCI=\frac{1-\left({\displaystyle\frac P{N+0.1}}\right)}{1-\left(\frac1{N+0.1}\right)}$, where P = total number of providers, N = total number of visits, n_i_ = number of visits per provider iAnalysed in quartiles with 1 = least continuous, 4 = most continuous 9) Usual Provider Continuity UPC index [73]: Ratio of the number of visits to the most frequently seen provider to the total number of visits to all providers 10) Duration of (established PCP-patient) relationship [79] 11) Rate of provider turnover [80]: the rate of a PCP leaves a clinical practice or retires 12) Most frequent provider continuity (MFPC): $MFPC=\frac{Max\left(n_1,n_2,\cdots,n_p\right)}N$. The proportion of outpatient visits that a patient has with his/her most frequent provider 13) Bice-Boxerman Continuity of Care [BB-COC]: $\frac{\left({\sum }_{i=1}p {n}_{i}^{2}\right)-n}{n(n-1)}$, where p = total number of providers n = total number of visits during episode ni = number of visits to provider i cj = indicator of sequential visits to same providers; equal to 1 if visits j and j + 1 are to the same provider, 0 otherwise [81] 14) Days out of PCP cover (DOC) were calculated by subtraction of the pre-defined optimal maximum time interval (updated according to diabetes severity level) from the actual time interval between a PCP service and the next healthcare service (either PCP or hospital admission) 15) Cover [82] shows a time-limited protective effect of primary care. The cover index = [∑ascertainment days—∑DOC] / ∑ascertainment days] was calculated for each individual annually. As the optimal maximum time interval was identified as a range of values from the threshold effects model, the cover index was calculated with low, middle and upper values bounds corresponding to low, middle and upper values of the optimal maximum time interval identified for each complication cohort
Regularity index [50, 51]: Measures how well distributed the PCP service utilisation is, not how often	1) Interval index [51] was based on with annual regularity of PCP contact defined as an ordinal variable with contacts over a 1-year period as none, any, annual, or semi-annual (at least one visit in each half of both years), or quarterly. For example, expressed as the percentage of PCP visits occurring in each quarter (0%, 25%, 50%, 100%) 2) Variance index [51]: $VI=\frac1{1+var\left(days\right)}$, where days are the number of days between consecutive PCP visits. Analysed in quartiles with 1 = least regular4 = most regular 3) Relative variance index (RVI): $RVI=1/\left(1+\left(\frac{sd\left(days\right)}{mean\left(days\right)}\times100\right)\right)$ where days are the number of days between consecutive PCP visits. Analysed in quartiles with 1 = least regular, 4 = most regular Differs from the variance index in that the coefficient of variation in the days between PCP contacts is used rather than the variance. At least two PCP contacts per year required to calculate. The modified index produces a unitless measure of variation, which is less correlated with frequency compared with previous measures [51]

### Identifying an individual’s likely responsible PCP

Researchers suggested that care coordination would improve when patients visit the same PCP for most of their primary care visits and when specialist referrals are determined by the likely responsible PCP [4, 10]. The issue of having multiple PCPs included increased medical services expenditures due to more office visits, prescriptions, and a more significant number of specialists seen for disease-specific populations [11]. Previous health services studies recommended that every patient see the same PCP [12]. However, in reality, patients may not have any responsible PCP, or they may have more than one. The claims-based “majority” and “plurality” algorithms (Table 1) were developed to identify the likely responsible PCP. The plurality provider is the provider who billed the highest number of Evaluation & Management (E&M) visits in one year, whereas the majority provider is the provider who billed for the plurality (greater than 50%) of all visits.

### Process measures

Several process measures have been documented in the literature, including medication intensification [13], lifestyle counseling documented in the notes of PCPs [14], the appropriate-care-model composite score for preventative care [15–17], time from patient discharge to the first visit with PCP [18, 19], peer review and identification of negligent adverse events, and the duration of PCP consultation [20](1). Rather than the manual extraction, peer review, and identifications from the PCP notes, the process of these face-to-face encounters can be efficiently extracted from appropriate billing codes. For instance, medication intensification, defined as the initiation of a new medication or an increase in the dose of an existing medication [21], can be identified from the pharmacy data. Morrison et al. [13] used the EHR data from Brigham and Women’s and Massachusetts General Hospitals to identify the acute encounters for acute pain and infection of patients based on the availability of appropriate billing codes and ICD-9 diagnosis codes. The data linkage to patients’ pathology test data for elevated A1C, lipid profile, or blood pressure [13] provided the possibility for identifying the preventative care services the patient received. The use of billing codes [22] may accompany great uncertainty and does not necessarily reflect every single patient's clinical information when the billing codes are created in a way more relevant to providers.

There was an ongoing debate regarding the optimal duration of the consultation. Orton and colleagues demonstrated that longer consultations had significant beneficial effects on patients [23]; however, the Cochrane systematic review by Wilson and Childs showed no incremental benefits from having longer consultations [24]. The controversy led to the development of new measures. The long to short consultation ratio [25, 26] was a method to measure the quality of care for general practices. The PCPs with the fastest times (1^st^ quartile) were assigned as “faster” doctors; those in the last quartile with the longest times were described as “slower” doctors; and within each of the three doctor styles, the percentage distribution of consultation lengths was displayed and the ratio of long to short consultations calculated. The billing codes for claims data [27] also allowed for identifying the duration of the PCP consultations. Since 2003, the Centers for Medicare & Medicaid Services (CMS) collected claims data for Medicare and Medicaid patients across various categories and times, including Inpatient and Outpatient claims, Master Beneficiary Summary Files, and many other files. CMS continues to update a list of Current Procedural Terminology (CPT®) and Healthcare Common Procedure Coding System (HCPCS) codes, bill procedures, and services codes, which the American Medical Association primitively developed. In CPT codes, 99,211, 99,212, 99,213, 99,214, and 99,215 corresponded to 5 ~ , 10 ~ , 15 ~ , 25 ~ , and 40 ~ minute consultation, respectively [27]. Since COVID-19, telehealth has experienced a dramatic increase. Connection to PCP via telehealth was not novel but emerged during the COVID period as a promising model of healthcare. Some measures, for example, “no-shows” and “same-day” cancellations, “after-hours” care availability (i.e., care that is not Mon–Fri, 8 a.m. – 5 p.m.) from both EHR and claims data have evolved as quality-based metrics [28].

Distance to the medical offices and availability of primary care services in patients’ communities are known barriers to access to care, especially in rural areas [29]. The geographic access estimation, for example, number of PCPs/10,000 population and availability of PCPs within a distance of a 30-min drive time [30, 31], enables the assessment of the spatial distribution of primary care providers and helps inform the health planners of the possibility of optimizing the health resources in order to achieve efficient primary care [32, 33].

### Continuity and regularity of care

Another core principle of primary care is the continuity of care [34]. It illustrates an ongoing relationship between the PCP and the patient beyond a single episode of illness. A couple of studies have found a strong correlation [35–38] of the continuity of PCP care with enhanced patient-physician relationships, which include trust-building, effective communications, a sense of responsibility over time, greater patient satisfaction and higher quality of care (e.g., better recognition of problems and diagnostic accuracy, effective management of patients with chronic conditions and maternity care outcomes), higher rates of compliance to medications, better performance of screening tests, timely receipt of preventive medicine services, less loss in follow-up visits, and a considerable reduction in hospitalizations, repeat hospitalizations, emergency department (ED) visits, and shorter length of hospital stay. There are multiple indices and algorithms to check the continuity of care from different dimensions (Table 1). From the review of existing evidence [39–42], we have found convergences and divergences in a considerable spectrum of continuity of care measurements with different considerations of what is essential to measure. For instance, they can be described as measures of concentration (the proportion of consultations with one specific provider), dispersion (the number of different professionals consulted), distribution (the distribution of consultations between providers, giving higher scores to people who consult fewer providers); or sequence (whether each consultation was with the same provider as the previous consultation) [43]. Some measures are based on attributing scores to individuals (“individual measures”), while others attribute a score to each consultation (“visit measures”). Some approaches have focused on visit patterns only, whereas others have defined an individual provider as the “primary” provider for each patient. Evidently, no single measurement approach could fully capture the whole concept. Therefore, more emphasis should be given to developing and applying direct measures from the patient’s perspective, focusing on information sharing and care consistency between various organizations, and broadening the inclusion of continuity measurement in the multi-disciplinary team level [41].

Continuity of care also has potential limitations. First, those measurements may not always have a straightforward meaning [43]; for example, the value between 0 (different doctors on every occasion) and 1 (all care from the same doctor) for the continuity of care (COC) index does not release a specific message by itself. Secondly, repeated contact with the same PCP may bring gain and loss. Having multiple PCPs [44] may allow confirmation of diagnoses and suggestion of additional directions for diagnoses after discussing with each other. Some British researchers shared their concerns that higher COC [45] might paradoxically impair the patients because familiarity may reduce the duration of each visit, prevent patients from discussing new problems, and lessen the PCP’s objectivity. It could frustrate PCPs [45] if their patients have insoluble issues, and the long-term continuity may lead to PCP burnout.

The temporal regularity of primary care visits has been a novel concept in recent years. This method of regularity score to assess the regularity of PCP was developed by Einarsdóttir et al. [46] as 1/[1 + Variance (${\varnothing}i$)], where ${\varnothing}i$ was the time interval between the (i-l)th and ith PCP visits. It ranged from 0 to 1 (with 1 representing perfect regularity) and was divided into quartiles, each containing 25% of the study population. Any individual score did not represent a unique distribution of any set number of PCP visits; instead, an aggregate tendency toward even spread and lack of clustering. The two hypotheses [47] behind the regularity index was (1) that a more temporally regular pattern of visits reflected the higher quality of care at some sites, achieved through more effective efforts to manage patients proactively and conversely to prevent loss to follow-up, and (2) temporal regularity was capturing unmeasured patient-level variables that are associated with risk for poor outcomes, such as a patient’s propensity to attend scheduled appointments or to participate in other health-promoting activities. Researchers were generally positive about this new measurement because they have identified a high correlation between high temporal regularity of PCP visits and positive health outcomes in various study samples  [47–51].

### Application: a case example for understanding the role of primary care in cancer survivorship

In this section, we present a case on operationalizing various metrics to guide the conceptualization and evaluation of emerging cancer survivorship care models involving PCPs [53, 54]. Foremost, the scope of the metrics can be compared against existing guidelines [55] or frameworks [56] to clearly define the role of PCPs. In the management of survivors of cancer, the relevant care domains include health surveillance, lifestyle modification, preventive care, management of physical, psychosocial issues, and chronic conditions, together with the use of survivorship care tools to ensure care continuity beyond the oncology to primary care setting, are highly compatible with the process and care continuity measures. It was postulated that a lack of regularity of interactions with the health system among cancer survivors compared to patients with other chronic conditions may have reduced opportunities for advocacy of self-management skills [57], a care gap that PCPs can address with existing skillsets [58]. Also, oncologists could conveniently apply algorithms to EHR data to identify the appropriate PCPs for survivors’ consideration, allaying reported concerns of disrupting existing doctor-patient relationships wherever possible [59, 60]. A clear delineation of the PCPs’ role could directly address existing divergent views reported in the literature [61] and demarcate roles and responsibilities [53] from tertiary providers. Furthermore, the clear links between these performance indicators and principles of primary care serve to reduce confidence-related hesitancy.

Besides helping to conceptualize PCPs’ roles, the library of measurements will allow a holistic and timely audit or evaluation of primary care involvement in new care models or programs using readily available EHR data in real time. By including metrics from different primary care aspects one can ensure comprehensive and robust outcomes to overcome the pitfalls of considering indicators in silo. Additionally, the granularity of the data from the metrics allows researchers to identify poor performing areas, facilitating the development of strategies to improve specific care aspects. Lastly, this case of cancer survivorship supported our recommendation on developing additional measures to assess information sharing and care consistency between various organizations. This endeavor will complement existing efforts by the American Society of Cancer Oncology to build oncology EHR for advancing and ensuring quality cancer care [62, 63].

### Challenges and opportunities

We acknowledge that, in some cases, the available data may not be ideally suited for the objective measures described above. The implementation of these quality measures requires substantial investments in data collection, processing, and analysis. The digital transition of medical records to EHRs requires tremendous efforts, which may not be immediately achievable at the local, state, and national levels. Most delivery systems rely on technical support provided solely by a single EHR software vendor and only identify the care process for network PCPs, which results in a lack of interoperability across providers, networks, and vendors. Additionally, the adoption of “big data” in healthcare in the United States falls behind other industries under the regulatory environment of the Health Insurance Portability and Accountability Act, and the Health Information Technology for Economic and Clinical Health Act enacted to protect personal health information in the United States [64], compared to some other countries (e.g., the United Kingdom), where the detailed electronic primary care records, procedure registries, cause-specific hospitalization, mortality record, and census data are available at a national scale [65].

Moreover, there was a knowledge gap between the data-based algorithms versus the patient-reported quality of PCP care. Bentleret et al. [66] compared the claims data-based COC indices with self-reported National Health and Health Services Use questionnaires of 2,620 Medicare beneficiaries and found that most claims-based COC measures failed to coincide with patients’ subjective perceptions of continuous patient-provider relationships. Future studies may potentially gain insight into the performance of the EHR and claims-based metrics.

The COVID-19 pandemic significantly impacts patient care and the healthcare system, including primary care. The pandemic has dramatically changed how primary care is delivered. Providers defer elective and preventive visits, such as annual physical exams, and convert in-person visits to telemedicine/telehealth visits to prevent person-to-person transmission. Currently, CMS [67] allows more telehealth services for all clinicians by expanding the terms of CPT codes, including remote evaluations, virtual check-ins and e-visits, and remote patient monitoring, and temporarily waive Medicare and Medicaid’s requirements that physicians and non-physician practitioners be licensed in the state where they are providing services. All of these efforts could take effect but leave many uncertainties about the factual delivery of primary care. As more clinical data become collected and standardized across providers, we hope that integrating EHRs and administrative claims data will be a cost-effective way to assess the quality of primary care.

## Conclusion

Population-level systematic assessment of the quality of primary care is challenging and costly. In the digital big data era, the integrated use of EHRs and administrative claims data sources may represent a promising cost-effective approach for comprehensive assessments of primary care quality. Migration to integrated universal health information infrastructure [83] and sharing and linkage of health data may support more systematic evaluation of primary care. However, significant efforts are needed to facilitate data integration in many countries. Bearing the limitation, machine learning and artificial intelligence have the potential to address some of the limitations mentioned in the context of population-level systematic assessment of primary care quality. Machine learning and artificial intelligence techniques can help in extracting valuable insights from the integrated use of EHRs and administrative claims data sources, which can support comprehensive assessments of primary care quality in a cost-effective way. However, there is a need for ongoing research and development to refine the existing measurements and develop new measures to assess all primary care domains effectively. Moreover, efforts are needed to facilitate data integration and ensure the security and privacy of health data.

## Data Availability

Not applicable.

## Abbreviations

PCP: Primary care physician
EHRs: Electronic health records
E&M: Evaluation & Management
CMS: Centers for Medicare & Medicaid Services
CPT®: Current Procedural Terminology
HCPCS: Healthcare Common Procedure Coding System
ED: Emergency department
COC: Continuity of care
